# Therapeutic setting as an essential component of psychedelic research methodology: Reporting recommendations emerging from clinical trials of 3,4-methylenedioxymethamphetamine for post-traumatic stress disorder

**DOI:** 10.3389/fpsyt.2022.965641

**Published:** 2022-11-03

**Authors:** Lauren Okano, Gregory Jones, Bri Deyo, Alida Brandenburg, Wesley Hale

**Affiliations:** ^1^Department of Training and Supervision, Multidisciplinary Association for Psychedelic Studies, Santa Cruz, CA, United States; ^2^Department of Psychiatry and Behavioral Sciences, McGovern Medical School, University of Texas Health Science Center at Houston, Houston, TX, United States; ^3^Department of Data Management, Multidisciplinary Association for Psychedelic Studies, Santa Cruz, CA, United States; ^4^Department of Research Development and Regulatory Affairs, Multidisciplinary Association for Psychedelic Studies, Santa Cruz, CA, United States

**Keywords:** psychedelic assisted therapy, MDMA, setting, research methodologies, recommendations

## Abstract

Research of psychedelic assisted therapies is at an all-time high, though few studies highlight extra-pharmacological factors that may affect treatment efficacy. One critical set of attributes includes the therapeutic setting itself, which describe the physical and socio-cultural environments in which the drug-assisted session occurs. Despite enduring consensus of the influence of setting, recommendations for establishing and reporting key setting variables remain sparse across clinical trial protocols and published research methodologies. The purpose of this paper is to: (1) present what is known of the influence and implications of setting to psychedelic-assisted therapies, with a particular focus on 3,4-methylenedioxymethamphetamine (MDMA); and (2) propose a set of reporting guidelines for operationalizing and reporting key setting variables in clinical trials of psychedelic-assisted therapies, based on recommendations emerging from clinical trials of MDMA for PTSD. In fact, recommendations should be expanded to “set” - the subject's mood, expectations, and broader psychological condition - once this is more fully developed in the field. The proposed reporting guidelines offer a means of increasing the volume and variability of data necessary for future empirical examination of key setting attributes influencing treatment efficacy, while preserving practitioner and patient autonomy to co-construct adaptive therapy settings according to their respective needs and expertise.

## Introduction

Research of psychedelic-assisted therapies is at a historical high (1), with 54% of the top-cited 100 psychedelic articles published in the last decade (2). Burgeoning scientific and political interest in psychedelic therapies – in particular for post-traumatic stress disorder (PTSD), addictions, anxiety, and depression – has led to the designation of psilocybin and 3,4-methylenedioxymethamphetamine (MDMA) as breakthrough therapies by the U.S. Food and Drug Administration (3–5). Further evidence of this increasing momentum is the Biden administration's recent call for top officials to prepare for the pending approval and regulation of MDMA and psilocybin-assisted therapies within the next 2 years in the United States (6). Thus, in anticipation of a rapid expansion of psychedelic clinical trials in the near future, there is now an ever urgent need for researchers to reflect on the past, present, and future status of clinical methodology and reporting in this space. Failure to do so could potentially result in the loss of invaluable data that could vitally enhance the long-term safety and efficacy of these medicines.

One such area of particular importance is reporting on the therapeutic setting, a variable which 5 describes as the physical and social contexts in which the psychedelic drug response unfolds, and which is especially relevant to psychedelic-assisted therapies due to the nature of current research protocols. Modern day clinical research protocols for psychedelic therapies involve a rigorous screening process, multiple non-drug preparatory psychotherapy sessions, single or multiple sessions with a psychedelic compound, and additional non-drug integration sessions (7). As the *psychedelic-assisted* moniker implies, rather than exerting solely pharmacological effects, this class of compounds is suggested to enhance psychotherapeutic processes when administered in a supportive setting (8–10). Conversely, lack of consideration for establishing a safe and supportive therapy setting has previously been associated with increased risk of adverse psychological reactions (11).

A recent literature review of 43 psychedelic therapy studies found that authors consistently highlighted the conceptual importance of the physical and social settings, but provided few and inconsistent details on the nature, operationalization, and/or hypothesized mechanisms by which specific setting attributes may affect treatment outcomes (12). Further, accelerating scientific and political interest in the therapeutic use of psychedelics (or perhaps in light of it), guidelines for reporting what constitutes a safe and supportive therapy setting remain sparse across clinical trial protocols and published research methodologies.

As psychedelic research continues to gain scientific and political traction, it is imperative to ensure valid and reliable inferences can be made to advance clinical practice and regulatory policies. Currently, due to the limited sample size of recent clinical trials and the lack of available data characterizing setting, it is not yet feasible to rigorously examine the direct significance of setting attributes to treatment outcomes. However, establishing guidelines for operationalizing and reporting these attributes as essential methodological variables is a prerequisite for future empirical research in order to test these hypotheses directly.

The purpose of this paper is to: (1) present what is known of the influence and implications of setting to psychedelic-assisted therapies, with a particular focus on 3,4-methylenedioxymethamphetamine (MDMA); and (2) propose a set of reporting guidelines for operationalizing and reporting key setting variables in clinical trials of psychedelic assisted therapies, based on recommendations emerging from clinical trials of MDMA for PTSD. While this paper focuses on setting, we acknowledge the importance of “set” – the subject's mood, expectations, and broader psychological condition – and intend to publish a future perspective article to complement this. Lastly, we acknowledge the lack of empirical evidence of key setting attributes specific to MDMA and propose these guidelines as a precursor to future experimental studies examining where settings best for MDMA differ from settings for classic psychedelics.

## Part 1a. What is known about how setting influences the response to psychoactive drugs

Indigenous cultures established the influence of environmental factors shaping the response to psychoactive substances (13) long before “set and setting” became common parlance among mid twenteeth century researchers (14, 15). Building on this, there now exists a number of natural and experimental studies widely cited as foundational evidence for the influence of physical and social environments on short and long-term responses to psychoactive drugs. This includes Robins et al. (16) and Zinberg (17) studies in which the use of heroin by soldiers serving in Vietnam was, on average, discontinued and did not evolve into addiction once service members returned to their safe and secure home - suggesting that secure and safe environments, or the lack thereof, play an important role in drug responses and addiction. This is further corroborated by Alexander et al. (18) seminal rat park experiment, in which rats housed in “social communities” with “comfortable bedding” were less likely to sustain cocaine use and overdose relative to rats kept solo in cages. Additionally, contemporary evidence involving both recreational MDMA users (19, 20) and clinical trial subjects (12, 21) consistently reinforce the influence of the surrounding environment on both subjective experiences and clinical outcomes of the individuals.

## Part 1b. Neurobiological and preclinical perspectives on the role of setting in MDMA-assisted therapy

The mechanism by which MDMA works synergistically with setting is not yet fully understood. Initial findings suggest it is partly attributable to its distinct synergy of psychostimulant (e.g. catecholaminergic enhancement), oxytocinergic, and classical psychedelic (e.g. 5-HT2A agonism) properties that increase sensitivity to internal (e.g., endogenous processes and pre-existing psychiatric conditions) and external (e.g., setting) attributes (22–24). Additionally, subsequent neurotrophic and neuroplastic downstream responses are thought to impart a “critical period,” during which altered awareness, interpretation, and integration of both endogenous (e.g., psychological) and exogenous (e.g., setting) stimuli can facilitate enhanced fear extinction, reward learning, and memory reconsolidation (23–25). Such effects may increase the tolerability of processing traumatic memories when administered in a secure, therapeutic setting designed to enhance and augment the effects of MDMA's pharmacodynamic profile (26–28).

Moreover, factorial analyses of psychometric scales to assess altered states of consciousness provide several clear delineations between MDMA and classical psychedelics that are relevant to setting, and which can inform future research design. Classical psychedelics (LSD and psilocybin), for example, appear far more likely to induce audio-visual, synesthetic, and imagery distortions whereas MDMA appears to impart a heightened state of blissful awareness (10, 22). It is thus plausible that “real” elements in the surroundings (e.g., sounds, artwork, lighting, and color) have distinct importance in the context of MDMA use because these sensory inputs are transmitted in a *heightened* fashion, rather than in a *restructured* fashion as seen with classical psychedelics. Thus, the optimal combination of sensory inputs to impart synesthetic, mystical-type experiences associated with classical psychedelic efficacy may be entirely different from those which can safely foster fear extinction and trauma reprocessing with MDMA.

Though these understandings remain preliminary, such hypotheses are highly testable (even *post-hoc*), if the appropriate data is provided. Such work is already underway with psilocybin (29). Cross-referencing validated experiential questionnaires with individual setting variables in future MDMA trials would appear a necessary preamble to experimentally disentangle drug x environment synergies.

## Part 1c. Setting in modern clinical trials of psychedelic assisted therapies

Despite ample emphasis on the conceptual importance of setting in studies of MDMA and other psychedelics, it is more common to omit, neutralize, or “control” for extra-pharmacological variables within the current model of controlled trials (13). Among the few placebo-controlled clinical studies that do specify key attributes of setting, the following have been documented as important methodological considerations: a quiet, protected environment with a living-room atmosphere, eye shades, headphones with instrumental music, creative imagery, soft lighting, a comfortable temperature, and soothing olfactory cues (8, 30–32). Of course, the concepts of comfort, creativity, and soothingness are all highly subjective, so the ability to adapt these aspects of the setting to each patient's preferences may be essential to maximizing therapeutic effect and minimizing adverse experiences. Baseline personality inventories may be helpful in this regard. Moreover, the social identities – especially visually-apparent aspects like race, gender, voice/language, and stature – of therapists and clinic staff also make up key aspects of the social and cultural context, given their known relation to trauma as well as the framing and interpretation of psychedelic experiences (33, 34). These aspects represent a vital nexus between set and setting which need to be explored.

One noteworthy exception to the dearth of verbiage dedicated to setting methodologies in MDMA trials is a study by Ot'Alora et al. (21), in which the authors cite and make available their treatment manual. The following attributes are specified in the methods section: lamps with “low glow,” curtains for privacy that “allowed natural light to come in so that participants could see the sky and tree tops,” “a couch that could be transformed into a bed,” largely instrumental playlists, plants, fresh flowers, end tables, upholstered chairs for the therapists, colorful rugs, paintings, a small desk and bookcase, and a safe for secure drug storage. This study is exemplary in its transparency and detailed methodologies pertaining to establishing an optimal therapeutic setting. It is clear that elements of setting differentially influence study participants based on their identities and lived experiences, and that efforts to personalize or customize may optimize drug effects, though further research is needed to disentangle the nature and magnitude of their respective influences (12).

## Part 2. Implications for research, policy, and practice

Given historical fallout from adverse events associated with psychedelics, setting optimization with regards to patient safety is particularly paramount. Serious medical adverse events (SMAEs) such as seizures, hyperthermia, hyponatremia, rhabdomyolysis, and serotonin syndrome have been associated with MDMA use, though not observed in clinical trials, and almost exclusively at higher doses in recreational settings (35). In contrast, current research demonstrates that serious medical adverse events (SMAEs) are exceedingly rare in patients taking MDMA in controlled settings [only one episode of ventricular extrasystole in six phase II and one phase III trials (*n* = 147)] (36). It is worth emphasizing that all these SMAE risks can be influenced by setting variables listed in Table 1, such as the quantity (and type) of fluid resuscitation given to patients, ambient temperature, lighting, availability of emergency medical equipment, and provider medical training.

**Table 1:** Reporting recommendations for documenting physical and socio-cultural setting variables in psychedelic assisted therapy studies.

**Physical setting attributes**		
**Attribute**	**Considerations/Definitions**	**Variable reporting recommendation**
Facility Location	The physical environment surrounding the treatment room. Describes the general feel of the site.	Facility Location (select one): __ Urban __ Suburban __ Rural Facility Type (select one): __ Hospital (Inpatient) __ Outpatient clinic __ Commercial space __ Residential-type space (Y/N) Do patients have the ability to choose between 2 or more locations?
Treatment Room	The room where the drug-assisted treatment session/s occur. Describes the general feel of the room and any built-in resources or features.	__Provide photograph or diagram of room features General Color Tones (select one): __ Mostly cool __ Mostly warm __ Mostly neutral Participant Seating (select all): __ Couch __ Futon (can be converted between couch and bed) __ Chair, recliner __ Chair, not recliner __ Bed Features (select all): __ Window __ Private bathroom __ Space for body motion (yoga, stretching, walking) (Y/N) Do patients have the ability to choose between 2 or more treatment rooms?
Accessories	Resources/comforting items available to the patient in the treatment room during the treatment. Describes items that exist in the space for a purpose other than aesthetics or clinical care. Does not include artwork.	Available (select all): __ Bowl or container for drug dispensation __ Eye shades __ Art supplies __ Journaling supplies __ Blanket __ Pillow __ Fan __ Fidget objects __ Musical instruments (Y/N) Do patients have the ability to self-select accessories?
Artwork	Treatment room decorations. Describes items that exist for aesthetics.	General Color Tones (select one): __ Mostly Cool __ Mostly Warm __ Mostly Neutral
		Imagery (select all): __ Nature __ Spiritual __ Religious __ Abstract __ Psychedelic Items (select all): __ Wall art, print __ Wall art, objects __ Statues, sculptures, figures (Y/N) Do patients have the ability to change or remove the art pieces that are displayed?
Lighting	The natural and created light inside the room. Describes the types and versatility of the light sources that are used during the drug-assisted session/s.	Light sources used (select all): __ Outside window, blackout curtains __ Outside window, light curtains or blinds __ Overhead lights, on/off __ Overhead lights, dimmable __ Floor lamps, on/off __ Floor lamps, dimmable __ Table lamps, on/off __ Table lamps, dimmable __ Nightlight __ String lights Dominant hue of lighting (select one): __ Warm, white-yellow __ Cool, white-blue __ Variable, i.e. LED changeable color bulbs (Y/N) Do patients have the ability to choose the amount and type of lighting used?
Sound	The ambient noise that can be heard from outside the treatment room or building, including phones ringing, people talking, dogs barking, and/or traffic noise. Describes unintentional exposure to sounds.	Exposure to sounds from outside the room (select one): __ No sounds __ Faint sounds, easy to miss __ Muffled sounds, able to ignore __ Identifiable sounds, can be disruptive (Y/N) Use of white noise machine
Music	Music is an emerging treatment modulator of great interest in psychedelic-assisted therapy research and as a treatment method of its own. Describes recorded songs or sounds that are played during the drug-assisted session/s.	Music delivery system (select all): __ Patient's own headphones __ High quality stereo headphones __ Earbuds __ Speakers __ Built-in surround sound
		Music playlist mostly… (select one): __ Instrumental, no vocals __ Instrumental with vocalizations that are not words __ Songs with words in patient's native language __ Songs with words in language patient does not speak Music playlist, volume, and delivery system are… (select one): __ Fully at patient's discretion during session __ Somewhat at patient's discretion but mostly set __ Fully set ahead of time, no ability to adjust during session
Scents	The natural or curated smell of the room. Describes which, if any, artificial scents are used and how air in the room stays feeling fresh.	Aromatic accessories (select all): __ None __ Scented candles __ Incense or herbs __ Essential oils __ Scented sprays (Y/N) Do patients have the ability to choose if or when scent is used?
Thermal Conditions	The ambient temperature of the room. Describes air handling and heating/cooling methods.	(Y/N) Central air handling (heating, cooling, circulation)? (Y/N) Temperature measured/kept Consistent? (Y/N) Do patients have the ability to control the thermostat in the room?
Food and Drink	The refreshment options that are available to the patient during the drug-assisted session. Describes types of food and drink, as well as storage and preparation options.	Available resources (select all): __ Refrigerator __ Electrolyte beverage __ Fresh fruits and/or vegetables __ Food prep space or counter __ Food prep sink (not in bathroom) __ Dishes/utensils (Y/N) Do patients have the ability to choose what they eat and drink throughout the dosing day?
Medical and/or Monitoring Equipment	The equipment that exists in the room for medical or other monitoring. Describes equipment that is present inside the therapy room due to research, safety, and/or other local protocols.	Equipment that is present and plainly visible (select all): __ Blood pressure machine __ ECG machine __ Video camera/s __ Microphone/s __ Automatic External Defibrillator (AED) __ First aid kit (Y/N) Does the participant understand that, while rare, a medical emergency could occur during their drug-assisted session?
Risk Minimization	Safeguards put in place to minimize the risk of serious medical adverse events (SMAEs)	(Y/N) Information on how to summon emergency assistance is easily accessible (Y/N) Windows are secured
		(Y/N) Electrical cords are wrapped and out of sight as much as possible (Y/N) Sharp objects (e.g. scissors, knives, etc) are either secured or removed (Y/N) Cleaning supplies are either secured or removed (Y/N) Automated External Defibrillator (AED) is available nearby
**Socio-Cultural Setting Variables**		
**Attribute**	**Considerations/Definitions**	**Reporting Guidelines**
Places	Describes the arrangement of the facilitators and participants relative to each other and the door	People Placement: __ Line (facilitators sit on either side of patient) __ Triangle (facilitators and participant equal distance from each other) __ Rectangle (facilitators close to each other, farther from patient) __ Backed up (one facilitators sits back from other therapist) Who is closest to the door? __ Patient __ Facilitator (Y/N) Does the participant have the ability to choose where everyone sits?
Physical Stature of Facilitator(s)	The physical appearance of a facilitator may be triggering to patients with specific person-related traumas. Describes some key aspects of physical stature that can be triggering.	Facilitator 1 (select all): __ Male __ Tall __ Overweight __ Muscular Facilitator 2 (select all): __ Male __ Tall __ Overweight __ Muscular (Y/N) Does the participant have the ability to choose their therapy pair?
Social Identities of Facilitator(s) and Patient	The social identities of the facilitator and the participant may trigger im-/explicit biases, which may impact trust and sense of safety. The process of establishing that trust is beyond the scope of setting. Describes the aspects of the social identities that are mainly visual and impact setting.	Facilitator 1 (select all): __ Gender: mostly masculine __ Gender: mostly feminine __ Gender: non-binary __ LGBTQ+ __ White racial/ethnic identity __ Non-white racial/ethnic identity: Please specify ________ Therapist 2 (select all): __ Gender: mostly masculine __ Gender: mostly feminine __ Gender: non-binary __ LGBTQ+ __ White racial/ethnic identity __ Non-white racial/ethnic identity: Please specify ________ Participant (select all): __ Gender: mostly masculine __ Gender: mostly feminine
		__ Gender: non-binary __ LGBTQ+ __ White racial/ethnic identity __ Non-White Racial/ethnic Identity: Please specify ________
Presence of Patient's Trusted Contact	The presence of a relative, close friend, or significant other before and/or after the drug-assisted session may increase feelings of safety and trust. Describes whether and when trusted contact was present	(Y/N) Did the patient request the presence of a trusted contact? Presence (select all): __ Before drug-assisted session __ After drug-assisted session (not overnight) __ After drug-assisted session (overnight stay)

Again, it is critical we understand how to optimize treatment efficacy while mitigating the risk of adverse effects (10). As exclusionary criteria and procedural rigor relax with more widespread use, these types of setting considerations will be of elevated importance. Risk of acute psychiatric complications (increasing depression, suicide, psychosis, and paranoia) seen with MDMA should also inform setting design and reporting (36, 37). In support of this, best practices derived from high-acuity facility design (close supervision, ligature minimization, securing elevated windows, removal of sharp objects, etc.) should be followed and documented in publications. Documenting these variables can not only serve as a checklist for clinicians to reduce short-term risk, but also help to promote widespread adoption of these safeguards by community practitioners through publication.

Detailed reporting of setting variables is also crucial for improving the external validity of psychotherapeutic techniques, study reliability, and comparisons of experimental data across investigations (38). Reliable neurobiological correlates of response remain elusive in psychiatry, in part due to inadequate accounting for extra-pharmacological variables. Psychedelic research presents a promising opportunity in that regard, as such variables are already a central focus of the therapeutic package itself. Emphasis on collection and dissemination of set and setting details, combined with the considerably larger effect sizes seen with psychedelics, may hold significant potential for robust biomarker development and further neuroscientific understanding. Thus, these data are foundational to future studies disentangling MDMA's mechanistic specificity, tracking and predicting therapeutic response, and isolating placebo effects - the basis for understanding how, when, for whom, and under what conditions therapies “work.”

Lastly, establishing expectations for transparent documentation of setting is essential in shaping more effective drug policy. In an effort to overcome sensationalism over tentative results, which has derailed prior waves of psychedelic research, scientists are increasingly being called upon to provide rigorous, detailed evidence to inform policy decisions and legalization frameworks (13, 39). Greater use of, and adherence to, reporting guidelines may ensure that findings can be synthesized, reproduced, and applied over time to optimize treatment and care (12). Looking to the future, it is vital that scientists, clinicians, and policymakers embrace this paradigm shift in understanding and utilizing drug x context synergies (8).

## Part 3. Reporting recommendations emerging from clinical trials and expanded access use of MDMA for PTSD

Table 1 presents reporting recommendations for the documentation and future empirical study of key therapeutic setting variables in psychedelic research, with a focus on MDMA therapy. These recommendations are based on the Multidisciplinary Association for Psychedelic Studies (MAPS) MDMA-Assisted Psychotherapy treatment manual (40), which was adopted from approaches to earlier psychedelic treatments (41–43), traditional psychotherapy (44), and modified for use with MDMA (20, 45). Additionally, these recommendations are informed by insights that emerged from semi-structured interviews of seven pioneering MAPS facilitators who refined this therapeutic approach across three phases of clinical trials of MDMA for PTSD and supervised multiple MDMA-assisted therapy training cohorts. These recommendations are primarily intended to guide documentation and reporting rather than suggest standardized protocol for organizing setting in practice; both trained staff and participants must be empowered to collaboratively leverage their respective experiences to construct optimal, adaptive therapy settings.

**Figure 1:**
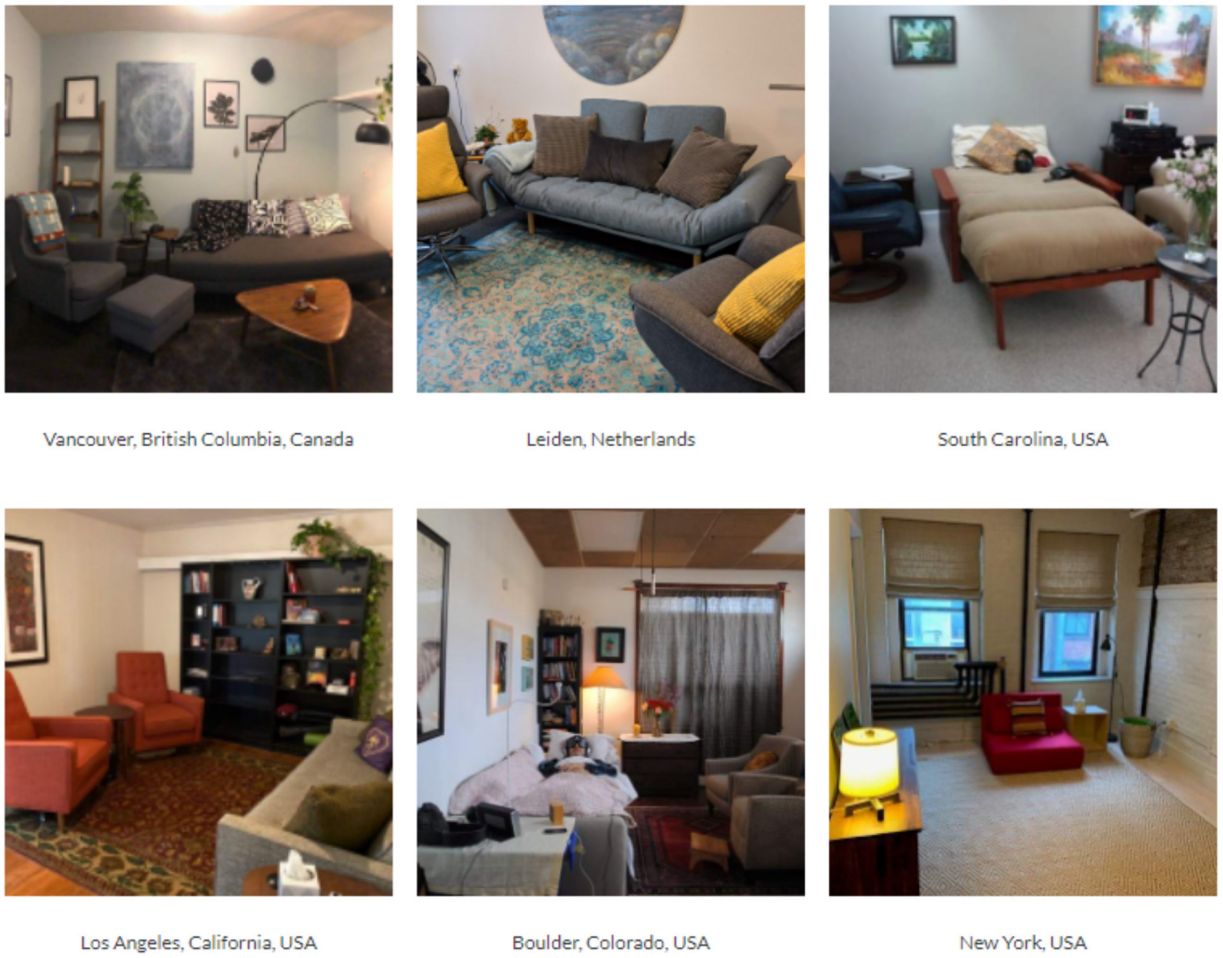
Photographs of MDMA-assisted therapy clinical trial settings.

We acknowledge the additional time and resources required to report these variables. However, we believe that doing so may ultimately avoid some reproducibility challenges in psychedelic science and ensure time and funding are efficiently expended. When possible, publication of pictures (e.g., room as a whole and individual elements), videos, playlists, or raw data as supplementary files can remove some of the burden from research staff. This may not only capture some unanticipated key elements, but can also serve as a repository of information to be mined by independent investigators. This may be especially helpful to those who have interest in psychedelic research, but who may not have access to clinical trials at their institutions. In addition to strengthening study validity and reliability, this increased transparency may also attract new investigators interested in conducting secondary data analyses. Figure 1 presents photographs of treatment rooms from MDMA-assisted therapy clinical trials as an exemplar of the significant variability which often goes unreported.

Ultimately, the primary consideration for any psychedelic-assisted therapy setting is to enhance patients' sense of safety and comfort, which makes paramount the documentation of how a setting was customized and adapted. Ensuring that setting attributes are free from connotations with the source of patients' trauma is of particular relevance to MDMA specifically. In the words of one pioneering MDMA therapist, “There isn't a right way. It must be attuned to the client's needs. Introducing and having a conversation about room elements and being curious about how they are received determines whether it is in service to the patient or dissonant with their experience.”

This notion is particularly relevant to documenting discernable attributes of the social environment surrounding the psychotherapeutic relationship – race, gender, sexual orientation, and stature of the patients and therapists – which are also inextricably linked to “set.” Cultural humility, relevance, and congruence are key to patients' sense of safety and trust within the therapeutic setting and therefore essential considerations to be documented in study protocols and methodologies (46). As another MDMA therapist describes, “If my stature or perceived identity creates anxiety in the room, then the participant's ability to access their authentic self is interfered with, decreasing safety and potential efficacy of the drug.” Hence, while further research is needed to disentangle the synergistic effects of both the physical and social attributes of setting, structured reporting of these variables is a seminal step forward.

## Conclusion

The use of reporting guidelines for documenting physical and social setting variables influencing psychedelic therapies may strengthen the rigor and reproducibility of research and treatment efficacy, while also strengthening regulatory efforts and mitigating risks to patients.

Further, future refinement of the recommended variables to report (Table 1) may offer a blueprint for inclusion in central study databases such as RedCap. While not exhaustive, these initial reporting guidelines offer a pragmatic step forward in increasing the volume of data around setting, offering parameters for reporting on study designs, and facilitating future study comparisons. Given the complexity of psychedelic research, these recommendations may not be universally applicable and are likely to evolve as the field progresses. Moreover, they are likely to vary based on the psychedelic drug, practitioner expertise, and patient need. Future research should examine the potential significance and modifying effects of the proposed setting attributes as they relate to treatment efficacy, and refine these reporting recommendations accordingly. We call on scientists to implement and iterate upon these guidelines to advance the medical, legal, and cultural contexts for people to benefit from the careful uses of psychedelics.

## Data availability statement

The original contributions presented in the study are included in the article/supplementary material, further inquiries can be directed to the corresponding author.

## Author contributions

All authors listed have made a substantial, direct, and intellectual contribution to the work and approved it for publication.

## Conflict of interest

Authors WH, BD, and AB receive full salary support from MAPS-PBC. The remaining authors declare that the research was conducted in the absence of any commercial or financial relationships that could be construed as a potential conflict of interest.

## Publisher's note

All claims expressed in this article are solely those of the authors and do not necessarily represent those of their affiliated organizations, or those of the publisher, the editors and the reviewers. Any product that may be evaluated in this article, or claim that may be made by its manufacturer, is not guaranteed or endorsed by the publisher.
